# Outstanding Reviewers for *RSC Advances* in 2024

**DOI:** 10.1039/d5ra90068a

**Published:** 2025-06-04

## Abstract

We would like to take this opportunity to highlight the Outstanding Reviewers for *RSC Advances* in 2024, as selected by the editorial team for their significant contribution to the journal.
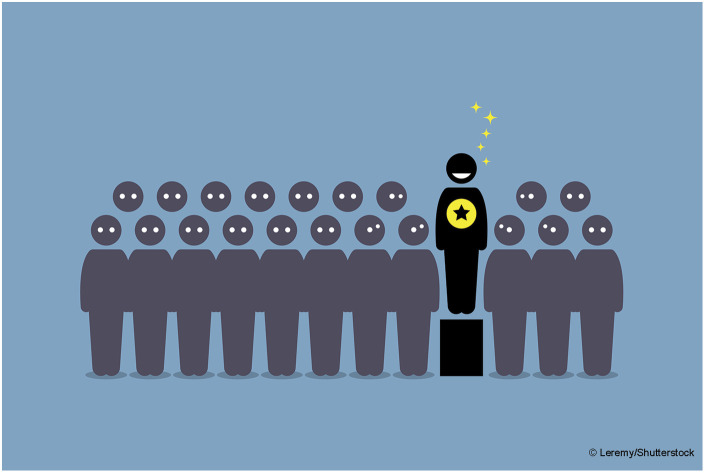

We would like to take this opportunity to thank all of *RSC Advances*’ reviewers for helping to preserve quality and integrity in chemistry literature. We would also like to highlight the Outstanding Reviewers for *RSC Advances* in 2024.

Each one of our outstanding peer reviewers has been carefully selected by our editorial team, and the list includes active researchers who have made significant contributions to peer review and have gone above and beyond in their actions. In recognition of the varied contributions of our reviewer community, our Outstanding Reviewers from 2024 have been chosen based on several different measures, including the number, timeliness, and quality of the reports completed during 2024. We also highlight reviewers who have provided exceptionally thorough and detailed reports, as well as reviewers who have made additional efforts to aid authors in improving their manuscripts.

We would also like to direct a special thanks to the members of the *RSC Advances* Reviewer Panel for their hard work and dedication, and the valuable contribution they have made to the journal. The Reviewer Panel is a key part of our commitment to deliver rigorous and fair peer review and ensures that manuscripts are handled by experts throughout the peer review process. We are proud to work with these individuals and recognise their crucial role for the journal.

*RSC Advances* strives to represent the diversity of the chemistry community, and we would like our reviewer community to reflect that. We are always looking to improve the diversity of our reviewer community in all its forms, and welcome applications for new reviewers through the Royal Society of Chemistry’s Become a Reviewer (https://www.rsc.org/journals-books-databases/author-and-reviewer-hub/reviewer-information/how-to-become-a-reviewer/) page.

In 2024 we introduced co-review to *RSC Advances*. Co-review enables early-career researchers to develop their skills in peer review through collaboration with an experienced colleague. It gives these researchers a chance to get reviewing experience, and to be recognised for their contributions. For more information on co-review, read our editorial: https://pubs.rsc.org/en/content/articlelanding/2024/ra/d4ra90099e

“Peer review lies at the heart of academic publishing. As a society journal we very much value the time, effort and expertise that our reviewers expend on our behalf. Excellent peer review ensures the academic quality of *RSC Advances* and so we extend our thanks to all those who have reviewed in the past year, and our special thanks to the group of excellent peer reviewers who have extended themselves even further on our behalf” – Russell J. Cox, Editor-in-Chief, *RSC Advances*

“Peer review is a vital aspect of ensuring the quality and originality of the research published in *RSC Advances*. Over the last year we are pleased to have launched co-review, which gives earlier career researchers the opportunity to gain experience and credit for taking part in the peer review process. Combined with transparent peer review, this will add to the quality of our peer review process as well as the transparency. We thank all the reviewers that continue to support *RSC Advances* and very much appreciate their contributions” – Karen Faulds, Editor-in-Chief, *RSC Advances*

***RSC Advances* 2024 Outstanding Reviewers:**

Dr Anindyasundar Adak

University of Birmingham

ORCID: 0000-0002-5955-4281

Dr Akbar Ali

Government College University Faisalabad

ORCID: 0000-0002-2914-0934

Dr Seemesh Bhaskar

University of Illinois at Urbana-Champaign

ORCID: 0000-0003-2714-3776

Dr Henrique de Castro Silva Jr

Universidade Federal Rural do Rio de Janeiro

ORCID: 0000-0003-1453-7274

Dr Yuan Fang

University of Virginia

ORCID: 0000-0003-1987-0885

Professor Alessio Gabbani

University of Pisa

ORCID: 0000-0002-4078-0254

Professor Lihua Gan

Tongji University

ORCID: 0000-0002-3652-8822

Dr Cemil Koyunoğlu

Yalova University

ORCID: 0000-0001-6309-1569

Dr Yixiong Lin

University of Utah

ORCID: 0000-0001-5769-5315

Dr Xin Lv

Zhejiang Normal University

ORCID: 0000-0002-5431-4529

Dr Luís Pinto da Silva

University of Porto

ORCID: 0000-0002-5647-8455

Dr R. Rani

Jain University

ORCID: 0000-0002-6116-1495

Dr Rüdiger Seidel

Martin-Luther-Universität Halle-Wittenberg

ORCID: 0000-0003-3438-4666

Dr Fateh Singh Gill

Graphic Era University

ORCID: 0000-0002-2726-6808

Dr Joonhyuk Suh

University of Georgia

ORCID: 0000-0002-3838-5861

Dr Zepei Tang

Montclair State University

ORCID: 0000-0002-9504-5812

Dr Kien Vu

Marian University, Indiana

ORCID: 0000-0002-1895-2224

Dr Masanori Yamamoto

Institute of Science Tokyo

ORCID: 0000-0001-8473-7015

***RSC Advances* Reviewer Panel 2024 Outstanding Reviewers:**

Dr Carlos Aiube

University of Brasília

ORCID: 0000-0003-2614-4363

Dr Elias Akoury

Lebanese American University

ORCID: 0000-0001-5202-8935

Dr Faiz Ali

University of Malakand

ORCID: 0000-0002-4984-164X

Professor Loai Aljerf

Damascus University

ORCID: 0000-0002-1132-9659

Dr Debmalya Bhunia

Cold Spring Harbor Laboratory

ORCID: 0000-0001-8649-0466

Dr Alien Blanco Fores

Technological of Superior Studies of Tianguistenco

ORCID: 0000-0003-4035-0550

Dr Olga Eremina

University of Southern California

ORCID: 0000-0002-2776-4743

Dr Serap Evran

Ege University

ORCID: 0000-0001-6676-4888

Dr S. Girish Kumar

RV College of Engineering

ORCID: 0000-0001-9132-1202

Dr Darrick Heyd

Toronto Metropolitan University

ORCID: 0009-0003-8195-4278

Dr Haradhan Kolya

Jeonbuk National University

ORCID: 0000-0003-3062-2321

Dr Feng Li

The University of Sydney

ORCID: 0000-0003-4448-074X

Dr Yu Li

North China Electric Power University

ORCID: 0000-0003-1430-7989

Dr Ekkehard Lindner

University of Tübingen

Dr Tharique Nalakath

MilliporeSigma Milwaukee

ORCID: 0000-0003-2245-5420

Dr Abhispa Sahu

SiOxMed LLC

ORCID: 0000-0002-3223-7577

Dr Kapil Upadhyaya

Oregon Health & Science University

ORCID: 0000-0001-9083-7784

Dr Fereshteh Vajhadin

University of Calgary

ORCID: 0000-0003-2361-8981

Dr Maria Vesna Nikolic

University of Belgrade Institute for Multidisciplinary Research

ORCID: 0000-0001-5035-0170

Dr Yan-Ping Zhu

Yantai University

ORCID: 0000-0003-1666-1850

We would also like to thank the *RSC Advances* Editorial Board and Associate Editors, and the chemistry community for their continued support of the journal, as authors, reviewers and readers.

We continue to work on improving the diversity of our reviewer pool to reflect the diversity of the communities that we serve.

Russell J. Cox, Editor-in-Chief, Leibniz Universität Hannover, Germany

Karen Faulds, Editor-in-Chief, University of Strathclyde, UK

Laura Fisher, Executive Editor, Royal Society of Chemistry, UK

